# Descemet membrane endothelial keratoplasty combined with secondary sulcus hydrophobic intraocular lens implantation

**DOI:** 10.1016/j.ajoc.2025.102399

**Published:** 2025-07-23

**Authors:** Colya N. Englisch, Philip Wakili, Clara E. Englisch, Karl T. Boden, Annekatrin Rickmann, Peter Szurman, André Messias

**Affiliations:** a Eye Clinic Sulzbach, Knappschaft Hospitals Saar, Sulzbach/Saar, 66280, Germany; b Department of Experimental Ophthalmology, Saarland University, Homburg/Saar, 66421, Germany; c Klaus Heimann Eye Research Institute (KHERI), Sulzbach/Saar, 66280, Germany; d Department of Ophthalmology, Otorhinolaryngology and Head and Neck Surgery, School of Medicine of Ribeirão Preto – University of São Paulo, Brazil

**Keywords:** DMEK, Pseudophakic ametropia, Hydrophobic, Piggyback IOL, Add-on IOL, Supplementary IOL, Secondary enhancement, Sulcus IOL, Polypseudophakia

## Abstract

**Purpose:**

To report a case of combined Descemet membrane endothelial keratoplasty (DMEK) and secondary sulcus hydrophobic intraocular lens (IOL) implantation for hyperopic correction in a pseudophakic eye with Fuchs’ endothelial corneal dystrophy.

**Observation:**

A 74-year-old woman with Fuchs’ dystrophy and a history of phacoemulsification with a hydrophilic IOL in her left eye (refraction: +5.25 D/–1.00 D × 68°; best corrected visual acuity [BCVA]: 20/40) underwent a preparatory yttrium aluminum garnet (YAG) iridotomy followed by implantation of a hydrophobic add-on +8 D IOL in the ciliary sulcus through a 2.4 mm incision. DMEK was performed using a 90 % fill of 10 % sulfur hexafluoride (SF_6_). Two months after surgery, refraction improved to −0.25 D/–1.75 D × 62° (BCVA: 20/25), with restored corneal clarity and an endothelial cell density of 1920 cells/mm^2^. At 10 months, refraction was +0.75 D/–1.25 D × 54°, and BCVA further improved to 20/20, with an endothelial density of 1778 cells/mm^2^ and normalized corneal thickness. No IOL opacification was observed.

**Conclusion and importance:**

This case indicates that combining DMEK with secondary sulcus hydrophobic IOL implantation can effectively correct hyperopia in pseudophakic eyes with Fuchs’ dystrophy.

## 1 Introduction

Fuchs’ endothelial corneal dystrophy is a common genetic disorder characterized by progressive deterioration of the corneal endothelium, affecting an estimated 300 million adults worldwide.^1^ As endothelial function declines, stromal edema develops, leading to visual impairment. Descemet membrane endothelial keratoplasty (DMEK) often fully restores vision and has become the standard of care for this condition.^2^

DMEK is a safe and effective procedure that delivers excellent graft survival and visual outcomes, and it can be performed either as a standalone surgery or in combination with cataract surgery and intraocular lens (IOL) implantation.^3^ However, eyes with a “complicated anterior segment”, including aphakic eyes, eyes with poor capsular support, eyes after glaucoma surgery, and vitrectomized eyes, exhibit significantly higher rates of graft failure.^4^ Additionally, performing DMEK in eyes with hydrophilic IOLs carries a significant risk of lens opacification.^5^ This complication is attributed to the interaction between the fixation gas and the hydrophilic properties of the IOL material, which can lead to deposit formation and reduced lens transparency.^6^

Various strategies have been investigated to address hyperopic refractive errors following cataract surgery.^7^ Although laser refractive correction remains a viable option, it is particularly challenging in hyperopic eyes with high diopter errors. Similarly, IOL exchange may be considered. However, its feasibility is often limited by the strong adhesion of the IOL to the capsular bag, a challenge that isfurther complicated when the posterior capsule has been opened using an yttrium aluminum garnet (YAG) laser. In eyes that subsequently require DMEK, the risk of inadequate capsular bag support after an IOL exchange becomes particularly significant. Alternatively, implanting a secondary “piggyback” IOL in the ciliary sulcus is a straightforward solution. In this case report, we describe a combined procedure involving DMEK and the implantation of a secondary add-on hydrophobic IOL in the ciliary sulcus.

## 2 Case report

A 74-year-old woman with Fuchs' endothelial corneal dystrophy was referred with symptoms of visual blurring. She had undergone combined phacoemulsification and DMEK in the right eye 6 months prior to referral and standalone phacoemulsification with hydrophilic IOL implantation in her left eye 7 years prior.

The refractive measurements were +0.75 D/–1.50 D × 120° in the right eye (BCVA: 20/20), but an unexpected +5.25 D/–1.00 D × 68° in the left eye (20/40).

**Fig. 1:**
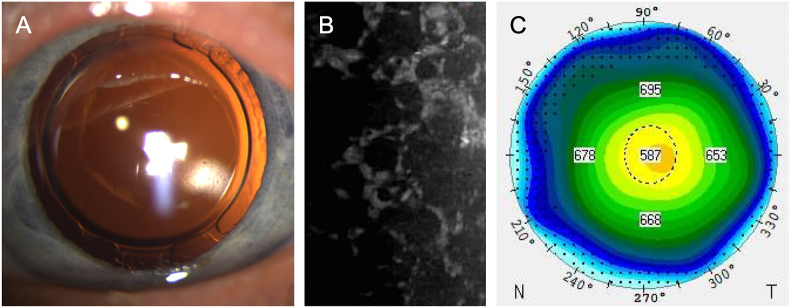
Preoperative examinations. Slit lamp photography (A), specular microscopy showing the typical Fuchs endothelial corneal dystrophy reduced cell density (B), and thickness map showing the increased corneal thickness (C, Pentacam, Oculus Optikgeräte GmbH, Wetzlar, Germany).

The right eye exhibited a clear cornea with an adherent DMEK graft and a well-positioned pseudophakic lens, while the left eye displayed post-YAG capsulotomy pseudophakia with corneal edema and guttae (Fig. 1). As expected, corneal specular microscopy showed a normal density of 1587 cells/mm^2^ in the right eye, but the endothelial density in the left eye was unmeasurable (Fig. 1). The vertex corneal thickness was normal (546 μm) in the right eye and increased to 590 μm in the left eye (Fig. 1).

The intraocular pressure (IOP) was 14 mmHg, and the results of the funduscopic examination, as well as macula and optic nerve optical coherence tomography (OCT), were unremarkable bilaterally. A surgical procedure combining DMEK with an additional acrylic hydrophobic three-piece monofocal IOL for implantation in the ciliary sulcus was scheduled. The IOL power calculation was performed using the Barrett Rx Piggy Back IOL Formula (https://calc.apacrs.org/barrett_rx105/) with a target refraction of −0.75 D using an IOLMaster 700 (Carl Zeiss Meditec AG, Jena, Germany).

After a preparatory YAG iridotomy performed the previous day, a +8 D IOL (MA60AC, Alcon) was implanted in the ciliary sulcus using the Alcon D cartridge through a 2.4 mm incision. DMEK was then performed as previously described, using 90 % fill with 10 % sulfur hexafluoride (SF_6_) for physiological IOP.^8^

At 2 months postoperatively, refraction in the left eye was −0.25/−1.75 × 62° (20/25). Slit lamp biomicroscopy and anterior segment OCT showed a well-centered and well-placed IOL. Gonioscopy did not reveal any trabecular pigment deposition. The anterior chamber depths were 4.49 mm and 4.05 mm in the right and left eyes, respectively. The left cornea exhibited a juvenile endothelial cell pattern and a restored density of 1920 cells/mm^2^, without any signs of corneal edema in the slit lamp biomicroscopy and pachymetry examinations. The vertex corneal thicknesses of the right and left eyes were 548 μm and 569 μm, respectively, with IOPs of 15 mmHg and 16 mmHg, respectively.

**Fig. 2:**
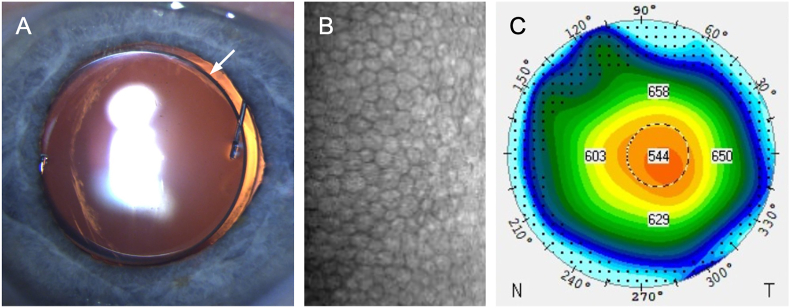
Postoperative examinations at 10 months. Slit lamp photography (A), specular microscopy showing normal cell density (B), and thickness map showing the normal corneal thickness (C, Pentacam, Oculus Optikgeräte GmbH, Wetzlar, Germany). The arrow indicates the secondary sulcus intraocular lens.

**Fig. 3:**
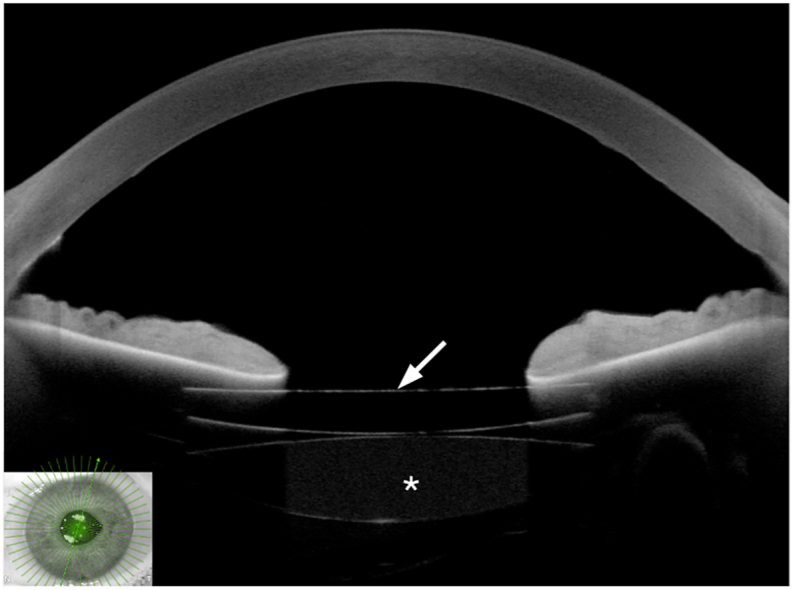
Anterior chamber optical coherence tomography showing the two intraocular lenses (IOLs) and their relationship with the iris. The arrow indicates the secondary sulcus IOL, and the asterisk indicates the primary IOL.

At 10 months postoperatively, refractions were +1.50/–1.50 × 119° (20/20) and +0.75/–1.25 × 54° (20/20) in the right and left eyes, respectively. The results of slit lamp biomicroscopy, gonioscopy, fundoscopy, keratometry, and anterior and posterior segment OCT were unchanged (Fig. 2, Fig. 3). In the left eye, the endothelial cell density amounted to 1778 cells/mm^2^, whereas the vertex corneal thickness decreased to 553 μm (Fig. 2). A similar reduction in endothelial cell density (1388 cells/mm^2^) was observed in the fellow eye at 10 months. The IOP was 17 mmHg in both eyes.

## 3 Discussion

This is a unique case reporting a combined piggyback IOL implantation with DMEK. The relatively long follow-up period indicates that this approach is both safe and effective, as showcased by the normalization of corneal thickness and endothelial cell count, as well as refraction correction from +4.75 to −0.13 D spherical equivalent.

The classical concept of piggybacking was introduced in 1993 by Gayton and Sanders, who aimed for a sufficient overall IOL power to treat a severe case of microphthalmos by implanting both the primary and secondary IOL in the capsular bag.^9^ However, classical piggybacking can lead to complications such as interlenticular opacification, capsular contraction, and the induction of a hyperopic shift.^10^, ^11^, ^12^ Today, secondary IOLs are more commonly implanted in the ciliary sulcus, an approach that has been proven to be efficacious, predictable, reversible, and overall safe. Nevertheless complications such as pigment dispersion, iris transillumination, pupil capture, elevated IOP, glaucoma, secondary endothelial injury, intraocular hemorrhage, cystoid macular edema, dysphotopsia, and hyperopic shift are conceivable, particularly when the implanted IOL was originally designed for capsular bag fixation.^11^, ^13^, ^14^, ^15^, ^16^, ^17^, ^18^, ^19^ In addition to monofocal models, bifocal and toric versions are available for various indications, including achieving multifocality in pseudophakic eyes with a monofocal primary IOL. Notably, Zimmermann et al. reported a case of secondary sulcus trifocal IOL implantation for presbyopia correction in an eye with previous DMEK, yielding satisfying results.^13^

Several issues were considered during surgical planning. It was anticipated that the anterior chamber might destabilize or narrow during the procedure, potentially compromising feasibility. However, no such issues were encountered, and graft unrolling and fixation with gas were performed without difficulty. Additionally, as indicated above, we were concerned that the piggyback IOL might increase postoperative inflammation, cause pigment dispersion, or elevate IOP. To date, none of these complications have been observed. Finally, if the hydrophilic IOL was to exhibit opacification following the combined DMEK and piggyback procedure, the removal of both IOLs might become necessary, which carries a risk of endothelial cell damage or lamellar detachment owing to anterior chamber manipulation.

An alternative approach would have been to implant supplementary IOLs specifically designed for the ciliary sulcus. However, these lenses are only available in hydrophilic material, which carries in turn a known risk of opacification.^20^

Importantly, in our case, no opacification of the primary hydrophilic IOL was observed up to 10 months, possibly because the hydrophobic piggyback IOL prevented direct contact between the gas and hydrophilic IOL surface. Indeed, hydrophilic IOL opacification is believed to result from direct exposure to air or gas, which triggers dehydration of the IOL material and subsequent crystal formation.^21^ Given the high rates of opacification with hydrophilic IOLs,^6^^,^^21^, ^22^, ^23^ implanting a hydrophobic IOL in the ciliary sulcus may be advisable—even in cases without refractive errors—for pseudophakic eyes with hydrophilic IOLs or an unknown IOL model scheduled for DMEK.

In this context, a new technique has been recently proposed to avoid hydrophilic IOL opacification after DMEK.^22^ Sise and Mekhail performed standard DMEK using a 90 % fill of 10 % SF_6_, but they suggested reducing the gas bubble before discharge so that the bubble's inferior margin cleared the superior pupillary border, avoiding contact with the IOL. This strategy resulted in a lower rate of IOL opacification than the standard technique.^22^ However, reducing gas-bubble volume at the first postoperative day might increase lamellar detachment risk, and even the short initial contact of gas with the IOL could cause IOL opacification in some cases.

An alternative approach involving IOL exchange with subsequent sulcus or scleral fixation, followed by DMEK either concurrently or in a staged procedure, may damage the zonules or further disrupt the capsular bag, particularly in cases such as the one presented here, where the posterior capsule is already impaired after treatment with the YAG laser. This could compromise ocular compartmentalization, increase the risk of gas leakage into the vitreous cavity, and ultimately jeopardize the fixation effect of the gas bubble on the DMEK graft, potentially affecting its adherence.

Moreover, IOL scleral fixation typically requires vitrectomy, which can make graft unrolling more challenging by hindering the ability to shallow the anterior chamber. In this setting, the "C-press" technique has been proposed to facilitate graft unrolling.^24^ However, it relies on corneal indentation, which may be difficult to reproduce consistently and could increase manipulation of both the anterior chamber and the DMEK lamella. Jacob and colleagues claim that glued intrascleral haptic fixation of a posterior chamber IOL successfully stabilized both the anterior chamber and the iris diaphragm–IOL complex, thereby enabling effective DMEK,^25^ while Gonnermann et al. reported favorable outcomes when performing DMEK in combination with posterior iris-claw aphakic IOL implantation.^26^ However, avoiding capsular bag disruption and vitreous manipulation using the piggyback approach appears to be a simpler alternative to the technique proposed in these studies. In addition, some surgeons use an anterior iris-claw-fixated IOL as a piggyback implant, avoiding the risk of pigment dispersion associated with a sulcus-fixated IOL. This is particularly useful in eyes with deep anterior chambers.

The case presented herein is unique in its combined application of a secondary sulcus IOL with DMEK for treating Fuchs’ endothelial corneal dystrophy. It indicates that DMEK can be successfully performed alongside sulcus IOL implantation in a single procedure to correct hyperopia in pseudophakic eyes, with highly satisfying clinical outcomes.

While longer-term follow-up is necessary to definitively confirm the absence of opacification in the hydrophilic IOL, it is important to note that the primary risk period, when intraocular air or gas may interact with hydrophilic materials, is typically limited to the early postoperative weeks. In our case, the implantation of a hydrophobic piggyback IOL over the pre-existing hydrophilic IOL may have mitigated direct exposure of the hydrophilic material to intracameral air or gas, potentially reducing the risk of opacification. However, we acknowledge that interactions between the two IOLs or other postoperative factors could contribute to late-onset opacification; therefore, ongoing monitoring is essential to fully assess the long-term outcomes.

## CRediT authorship contribution statement

**Colya N. Englisch:** Writing – original draft, Visualization, Investigation, Data curation, Conceptualization. **Philip Wakili:** Writing – review & editing, Visualization, Data curation, Conceptualization. **Clara E. Englisch:** Writing – review & editing, Visualization, Data curation. **Karl T. Boden:** Writing – review & editing, Project administration, Conceptualization. **Annekatrin Rickmann:** Writing – review & editing, Conceptualization. **Peter Szurman:** Writing – review & editing, Project administration, Conceptualization. **André Messias:** Writing – review & editing, Methodology, Investigation, Conceptualization.

## Declarations and patient consent

The study adhered to the tenets of the Declaration of Helsinki. Anonymous case reports are waived by the local Institutional Review Board (Ethikkommission bei der Ärztekammer des Saarlandes). The patient formally assigned the written consent for publication.

## Authorship

All authors attest that they meet the current ICMJE criteria for Authorship.

## Funding

No funding or grant support.

## Declaration of competing interest

The authors declare that they have no known competing financial interests or personal relationships that could have appeared to influence the work reported in this paper.

## List of abbreviations

BCVA: best corrected visual acuity; DMEK: Descemet membrane endothelial keratoplasty; IOL: intraocular lens; OCT: optical coherence tomography; SF_6_: sulfur hexafluoride; YAG: yttrium aluminum garnet.
